# Pilot Study of Attitudes of Taiwanese Veterinarians and Undergraduate Veterinary Students toward Animal Abuse and Interpersonal Violence

**DOI:** 10.3390/ani12091135

**Published:** 2022-04-28

**Authors:** Yi-Hsuan Chen, Wei-Hsiang Huang

**Affiliations:** 1 Department of Veterinary Medicine, School of Veterinary Medicine, National Taiwan University, Taipei City 10617, Taiwan; b05609074@ntu.edu.tw; 2 National Taiwan University Veterinary Hospital, College of Bioresources and Agriculture, National Taiwan University, Taipei City 10672, Taiwan; 3 Graduate Institute of Molecular and Comparative Pathobiology, School of Veterinary Medicine, National Taiwan University, Taipei City 10617, Taiwan

**Keywords:** animal abuse, animal cruelty, interpersonal violence, veterinarians, education, animal welfare, forensic science

## Abstract

**Simple Summary:**

There is a strong association between animal abuse and interpersonal violence; therefore, veterinarians may encounter both. Dealing with animal abuse cases is beneficial for advancing animal welfare and the overall public health. Veterinarians play an important professional role in identifying and responding to this relationship. This is the first study in Taiwan to investigate the current situation of animal abuse from the perspective of veterinarians. Our results established that the incidence of suspected physical animal abuse encountered by veterinarians in Taiwan was 0.16 cases per 100 patients, and 63.9% of our respondents had seen suspected of animal physical abuse in the past five years. Approximately 10% of animal abuse cases were likely concurrent with human abuse. Most respondents (about 80%) agreed that animal abuse and human abuse frequently co-occur. However, it did not affect their willingness to assist or report abuse. In total, 88.7% of respondents supported mandatory reporting of animal abuse. The results of this study underscore the urgent advancement of education and the importance of the crucial veterinary role in both animal and interpersonal abuse cases.

**Abstract:**

There is a strong association between animal abuse and interpersonal violence; therefore, veterinarians may encounter both. Dealing with animal abuse cases is beneficial for advancing animal welfare and the overall public health. Veterinarians play an important role in identifying and responding to this relationship. This study estimated the incidence of animal abuse encountered by veterinarians, examined veterinarians’ awareness of the relationship between animal abuse and human abuse, examined veterinarians’ attitudes towards how they deal with abuse cases, and related demographic characteristics to their attitudes of intervention and the frequency of encountering abuse cases. An anonymous self-administered questionnaire was designed and distributed through social media. Our results show that respondents’ motivation to interfere for animal abuse cases was positively related to their moral or legal responsibility, willingness to assist, and agreement of mandatory reporting. Our results indicated that respondents who believed they had been provided with adequate training were more willing to deal with animal abuse, more capable of distinguishing abuse cases, and did not believe that dealing with abuse cases was beyond their ability. However, more than 60% of our respondents self-evaluated that the animal cruelty awareness training courses were insufficient. Hence, in addition to the traditional role of veterinarians, identifying and responding to animal cruelty should be enhanced through education.

## 1. Introduction

There is a strong association between animal abuse and interpersonal violence; therefore, veterinarians may encounter both animal abuse and interpersonal violence clinically [1]. Traditionally, veterinarians care for animal health. However, animal abuse and interpersonal violence will affect each other and sometimes cannot be separated. Therefore, dealing with animal abuse cases by veterinarians positively contributes to the overall public health. Veterinarians play a crucial role in identifying and responding to this link. There is still a lack of research on the association between animal abuse and interpersonal violence from a veterinary perspective in Taiwan. The current situation, i.e., the incidence of animal abuse cases faced by clinical veterinarians in Taiwan, is unknown. Other information, such as the veterinarians’ attitudes towards abuse cases, the difficulties they may encounter, the extent of background knowledge (the knowledge of animal abuse and the understanding of legality), and awareness of the correlation between animal abuse and interpersonal violence, is lacking. Therefore, it is necessary to collect and analyze these data.

Animal abuse is an unkind behavior towards animals and an intentional act that harms animals [2]. In Taiwan, the Animal Protection Act (since 1998, last amended in 2021) states that animal abuse refers to “harming an animal or making it unable to function properly with violence, drug diversion, physical objects, acts of omission, or other means, beyond what is necessary to rear, tend or dispose of an animal” [3]. There are various other definitions of animal abuse; one of the most cited is “a socially unacceptable behavior that intentionally causes useless pain, suffering or anguish to the animal, and/or its death” [4]. Animal abuse can be categorized based on the type of abuse and intent to abuse. Companion animal abuse is classified into physical and mental types, and both include intentional and non-intentional maltreatment [5].

Animal abuse is a part of the spectrum of family and community violence and should be viewed as a leading public health problem worldwide [6]. Extensive research has linked animal abuse with interpersonal violence and even used it as an indicator and/or predictor of ongoing crimes of interpersonal violence and public health problems [7]. The earliest article in which this concept was proposed could be traced to McDonald’s triad of behaviors in children that appeared to be predictive of violence: enuresis, fire setting, and cruelty to animals [8]. In 1987, the American Psychiatric Association first included “physical aggression to people and animals” in the Diagnostic and Statistical Manual of Mental Disorders (DSM-III) as one of the earliest and most severe symptoms of a Conduct Disorder.

A review of human and animal service professionals stressed strong associations between domestic violence, child abuse, and animal abuse, proposing that it is the responsibility of both animal service and human service professionals to be aware of such occurrences, understand the significance, and promote appropriate professional and policy responses [9]. As empirical research demonstrates a close relationship between animal abuse and crimes against humans, the legal consequences of animal abuse have been strengthened. By 2014, all 50 states in the United States regarded animal cruelty as a felony. In 2016, the US Federal Bureau of Investigation began tracking animal cruelty crimes using the National Incident-Based Reporting System (NIBRS) [10].

Veterinarians play an important role in early identifying animal abuse and even interpersonal violence. However, most veterinarians are not trained to intervene in cases of animal abuse and interpersonal violence. Therefore, veterinary medicine schools should increase the amount of time devoted to animal cruelty issues in their curricula and offer online and on-site postgraduate and continuing education to build confidence in reporting animal cruelty [9].

In addition to education, policy protection should be provided for assisting veterinarians in solving ethical dilemmas. When veterinarians encounter such animal abuse cases, it is difficult for them to balance economic, safety, confidentiality, legal, and management concerns with ethical principles, personal beliefs, and professional standards [11]. While veterinarians remain divided on whether to report suspected abuse, Randour et al. suggested that all states in the United States should mandate that veterinarians report animal cruelty and offer immunity for good faith reporting [9]. The American Veterinary Medical Association considers it the responsibility of the veterinarians to report acts of animal cruelty and educate clients regarding humane care and treatment of animals. Currently, there is no mandatory reporting expected of veterinarians about animal abuse in the United Kingdom. In the United States, 20 states place a mandatory duty upon state-licensed veterinarians to report suspected animal cruelty; other states do not require veterinarians to report but rather allow veterinary professionals to act at their discretion. About 14 states have no laws that allow or require reporting [12]. In Taiwan’s Animal Protection Act or Veterinarian Act, there is no requirement to report any suspected or confirmed cases of animal abuse by veterinarians.

Considering the veterinarian’s awareness of the link between animal abuse and interpersonal violence, according to Monsalve et al., 2017, only 4.2% of the 96 published articles discussing this link were from Asia, i.e., China, India, Japan, and Malaysia [13]. In total, 79.2% of the studies were concentrated in North America, suggesting that this “link” remains unknown in various countries. In addition, only 7.3% of the articles were published in the field of veterinary medicine. Since Taiwan’s Animal Protection Act has been amended several times in recent years, we wish to further enhance the awareness of animal protection in veterinary medicine. In Taiwan, there are currently no published data regarding the incidence of veterinarians identifying human or animal abuse. Therefore, we designed a questionnaire, which is a modified version of the survey in New Zealand [14], aimed at investigating animal abuse cases encountered by veterinarians and the attitudes of veterinarians on issues of suspected animal abuse, interpersonal violence, and the link between these factors in Taiwan.

This study aims to (1) estimate the incidence of suspected animal abuse encountered by veterinarians, (2) study veterinarians’ awareness of the relationship between animal abuse and human abuse, (3) explore the attitudes of veterinarians towards dealing with abuse cases, and (4) relate demographic characteristics to their attitudes of intervention and their frequency of encountering abuse cases.

## 2. Materials and Methods

### 2.1. The Questionnaire and Data Collection

The questionnaire applied in this research was a modified version of the survey in New Zealand [14], which was based on similar research in Australia [15]. We revised this questionnaire to accommodate Taiwanese culture and language.

Before data collection, this questionnaire was first reviewed by 15 selected veterinarians to rectify inappropriate sentences and avoid meaningless answers.

An anonymous Google form link of our questionnaire was then created and distributed through the School of Veterinary Medicine, National Taiwan University’s Facebook page, and the Taiwanese Veterinary Medical Association’s website.

The questionnaire consisted of five sections (see Appendix A).

Section 1: Demographics. Demographic information was collected, including gender, age group, year of graduation, type of clinical practice, and number of monthly visiting clinical cases. Multiple choices were allowed to be selected for “type of clinical practice.”

Section 2: Attitudes. This section focused on the attitudes of veterinarians in facing abuse cases. In this section, the respondents would answer “how they would deal with abuse cases”, “what is their perception of the relationship between the perpetrator and other interpersonal violence”, and “whether veterinary education provides relevant knowledge.” The respondents were asked to rate their answers of 10 questions using the 6-point Likert scale from 1 to 6, with 1 = strongly disagree, 2 = disagree, 3 = slightly disagree, 4 = slightly agree, 5 = agree, and 6 = strongly agree.

Section 3: Managing Abuse. In this section, the questions included “if they believed they could identify abuse or not”, “whether they would choose to report and/or to assist when they encounter suspicious animal and/or human abuse cases”, and “What are the consequences they worry about when dealing with abuse cases?” In addition, opinions regarding “it should be mandatory to report animal abuse and human abuse” were asked.

Sections 4 and 5: Physical and mental animal abuse. These two sections focused on physical and mental animal abuse, respectively, and were limited to veterinarians with clinical experience. In the beginning, the respondents were asked again, “if they believe they have the knowledge to identify suspected animal abuse”. Other questions included “the frequency of suspicious animal abuse cases they had encountered within five years”, “if there was any interpersonal violence involved”, “who brought the animal to the clinic?”, “why it was suspected to be caused by abuse?”, and “what was the species of animal?”. Multiple choices were allowed in “what was the species of the animal?”.

Data were collected from 15 October 2020 to 30 November 2020. A total of 247 responses were obtained during this period. This questionnaire was conducted with absolute anonymity, and it was ensured that all the data including personal identifiable information collected and analyzed in the study complied with the country’s privacy and security laws. All responses were collected with the consent of respondents.

### 2.2. Statistical Methods

Descriptive statistics and frequency distributions were computed using SPSS (SPSS Statistics, IBM Corp., Somers, NY, USA). Likert-scale questions with ranking data were analyzed using Spearman’s rho between questions to interpret their correlation. A correlation coefficient between 0 and 0.3 is defined as low, a correlation coefficient between 0.3 and 0.7 is moderate, and a correlation coefficient between 0.7 and 1 is high. One-way analysis of variance (ANOVA) was used to compare the mean ratings of the Likert-scale questions with categorical variables. Chi-square tests were used to compare categorical variables.

## 3. Results

### 3.1. Section 1: Demographics

Details of the demographic and practice characteristics of the respondents are summarized in Table 1.

A total of 247 respondents completed this questionnaire, of which 53.8% (*n* = 133) were practicing veterinarians. The definition of practicing veterinarians excludes those who will graduate after 2021 and those who chose “none” in the number of visiting cases per month in this study. Sections 4 and 5 of the questionnaire were only filled by practicing veterinarians. Among these practicing veterinarians, most were female (59.4%, *n* = 79), while a minority were male (40.6%, *n* = 54). In 2020, there were 5486 veterinarian practice licenses in Taiwan [16]. The practicing veterinarians in this questionnaire are estimated to account for 2.42% of veterinarians in Taiwan.

The largest proportion of respondents (65.2%, *n* = 161) comprised those aged 18–29 years. Veterinary student respondents, whose graduation year was after 2021, accounted for 40.5% (*n* = 100). Among veterinarian respondents, the largest proportion had graduated between 2016 and 2020 (24.3%, *n* = 60).

In terms of clinical practice type, most veterinarians worked with dogs and cats (74.1%, *n* = 183). The second largest group of veterinarians worked with small animals, such as rabbits and guinea pigs (21.5%, *n* = 53). Other optional responses filled in by respondents included veterinary pathologist (*n* = 3), laboratory animal veterinarian (*n* = 2), horse veterinarian (*n* = 1), and animal protection officer (*n* = 1).

### 3.2. Section 2: Attitudes

#### 3.2.1. Comparison among Items

The attitudes of veterinarians when facing abuse cases are shown in Table 2. Items 1 to 3 were questions about attitudes related to interventions for animal abuse, and there was a noticeable trend that the higher the score, the greater the proportion among the three items. For item 1, 42.3% strongly agreed that the veterinarian has a moral or legal responsibility to intervene in suspected animal abuse, and the mean score was 5.05. For item 3, 45.6% strongly agreed with, “In practice, when presented with an animal abuse case, I would assist in preventing it from happening again,” and the mean score was 5.17. However, in item 2 “In practice, when presented with a suspected animal abuse case, I know my legal rights and responsibilities”, although most veterinarians were aware of their legal rights and responsibilities, only 28.6% of them strongly agreed this; scores in item 2 appeared to be more evenly distributed than item 1 and item 3, with a mean score of 4.28.

We analyzed the results of these three items using Spearman’s rho to determine whether a correlation between the respondents’ choices existed. The analysis showed that they were all moderately correlated with each other. The correlation coefficient between items 1 and 2 was 0.507 (*p* < 0.01). Likewise, the correlation coefficient between items 1 and 3 was 0.585 (*p* < 0.01), and the correlation coefficient between those who agreed in items 2 and 3 was 0.455 (*p* < 0.01).

Item 7 evaluated the attitude of veterinarians regarding the relationship between “training animals using punitive methods” and “animal cruelty.” The highest rate (28.2%) of responses was for point 4 (slightly agree) on the scale, with a mean score of 4.14.

Items 4 to 6 were questions about whether the perpetrators of animal abuse were more likely to be involved in other crimes. Surprisingly, the answers to these three questions were highly consistent: more than 40% of the respondents strongly agreed that people who abuse animals are more likely to commit other types of crimes, including interpersonal violence. The highest percentage (47.6%) of respondents who strongly agreed was in item 4 regarding child abuse, with a mean score of 4.92. The correlation coefficient between items 4 and 5 was very high (0.876, *p* < 0.01).

Items 9 and 10 were questions about attitude toward intervention in interpersonal domestic violence. Item 9 explored, “when domestic violence is suspected, the veterinarian has a moral responsibility to intervene.” Although the mean score was 3.85, the majority of respondents (27.4%) selected point 4 (slightly agree). In addition, the score was significantly lower than that for intervention for animal abuse (item 1, mean score = 5.05). Item 10 explored, “when presented with child or spousal abuse, I feel I should provide assistance to the client.” Compared to item 9, the mean score of item 10 was higher (mean score = 4.23), and the largest proportion of respondents (25%) selected point 5 (agree).

The correlation coefficient between item 10 (“in practice, when presented with child or spousal abuse, I feel I should provide assistance to the client”) and item 3 (“in practice, when presented with an animal abuse case, I would assist in preventing it from happening again”) was 0.420 (*p* < 0.01), indicating a moderate correlation. The mean score for item 10 was lower than that for item 3.

Furthermore, the correlation coefficient between item 10 (“in practice, when presented with child or spousal abuse, I feel I should provide assistance to the client”) and item 4 (“people who abuse animals are more likely to abuse their children”) was 0.294 (*p* < 0.01). The correlation coefficient between item 10 (“In practice, when presented with child or spousal abuse, I feel I should provide assistance to the client”) and item 5 (“People who abuse animals are more likely to abuse their spouse”) was 0.303 (*p* < 0.01). These results revealed low correlations.

Finally, item 8 explored, “during veterinary training, I was provided with adequate information and training to identify and prevent animal abuse”. The mean score was 3.07, and the majority of respondents (26.2%) selected point 2 (disagree). The correlation coefficient between item 8 (“during veterinary training, I was provided with adequate information and training to identify and prevent animal abuse”) and item 2 (“In practice, when presented with a suspected animal abuse case, I know my legal rights and responsibilities”) was 0.412 (*p* < 0.01), indicating a moderate correlation.

#### 3.2.2. Differences among Demographic Categories

Table 3 shows a comparison of mean scores of selected items (items 3, 4, 5, 8, and 10) among demographic categories, including gender, age, and year of graduation.

The comparison of scores for items 4 and 5 (the veterinarian’s attitude toward the recognition that animal abusers are more likely to abuse a child or spouse) showed only a borderline significant difference (*p* = 0.052) for age group in item 5. There were no significant differences for gender, age, or year of graduation.

The comparison of scores for items 3 and 10 showed only a borderline significant difference (*p* = 0.064) for age group in item 3. There were no significant differences for gender, age, or year of graduation. In terms of whether veterinarians will choose to intervene for animal abuse cases (item 3), the 18–29 age group was the most willing to assist, while the 40–49 age group was the least willing to assist.

The comparison of scores for item 8 showed significant differences among graduation year groups (*p* < 0.01). Veterinary students who will graduate after 2021 agreed more with “adequate information and training”. By contrast, veterinarians who had graduated in 2011–2015 agreed less with this statement.

We further analyzed the opinions of veterinary students expected to graduate after 2021 and veterinarians who had graduated before 2020 on these issues and found significant differences, mostly relating to items 3 and 8 (*p* < 0.01 for both). It was verified again that students who had not yet graduated agreed more with “adequate information and training”, and the responses further indicated that they were more willing to provide assistance for animal abuse.

### 3.3. Section 3: Managing Abuse

#### 3.3.1. Willingness to Report Abuse Cases

In this section, respondents were asked to indicate their willingness to report suspected animal and human abuse. Of the 247 respondents, 122 (49.4%) respondents chose to report all cases, 114 (46.2%) chose to report only severe cases, and 11 (4.5%) chose not to report when they encountered suspected animal abuse cases. In suspected human abuse, 115 respondents (46.6%) chose to report all cases, 107 (43.3%) chose to report only severe cases, and 25 (10.1%) chose not to report.

Table 4 shows the mean scores of selected items in Section 2 of the questionnaire in Appendix A, among groups for the willingness to report suspected animal and human abuse.

For item 1, concerning the attitude toward the veterinarian’s responsibility to intervene in suspected animal abuse, respondents who chose not to report animal abuse had a lower mean score (4.00 ± 0.77 for animal abuse and 4.44 ± 1.04 for human abuse). By contrast, respondents who chose to report all cases had a higher mean score (5.41 ± 0.81 for animal abuse and 5.28 ± 1.01 for human abuse). The *p* values among these were all <0.01, indicating that the more likely a veterinarian was to report abuse cases, the higher the moral or legal responsibility they felt toward animals.

With respect to item 9, concerning whether to intervene in suspected interpersonal domestic violence, the mean scores of those who chose not to report were very low (2.45 ± 1.29 for animal abuse and 2.44 ± 1.16 for human abuse). Even the respondents who chose to report all cases had relatively lower mean scores (4.20 ± 1.41 for animal abuse and 4.40 ± 1.36 for human abuse) compared with item 1. There was a similar trend for item 1, and the *p* values among these were all < 0.01.

Similar trends were observed for items 3 and 10. Irrespective of animal abuse or child/spousal abuse, the more likely the respondents were to report, the more they agreed to assist in abuse cases.

For items 4 and 5, all mean scores were greater than 4; however, there was no significant difference between whether they chose to report abuse or not.

#### 3.3.2. Concerns and Obligations Associated with Reporting Abuse Cases

**Figure 1:**
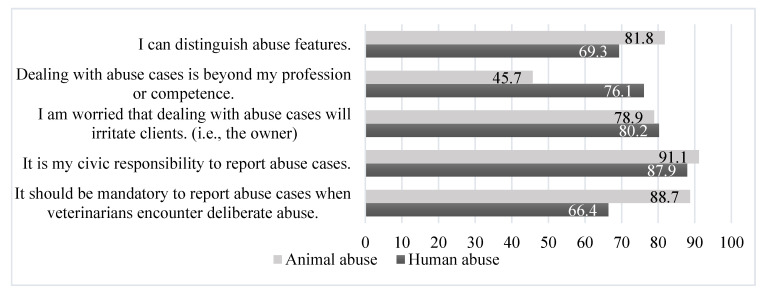
Percentages of respondents who responded to statements about how they would deal with abuse cases.

Respondents were then asked to indicate how they would respond to potential abuse cases in the clinic. The results are shown in Figure 1. Cross-analyses were performed based on the results. Table 5 shows the mean score of item 8 of Section 2 of the questionnaire in Appendix A, i.e., “during veterinary training, I was provided with adequate information and training to identify and prevent animal abuse”, among the groups concerning the reporting of suspected animal and human abuse. Table 6 shows the cross table between each concern and obligation (i.e., whether it should be mandatory to report abuse cases). Table 7 shows a cross table between obligation and demographic categories.

When asked, “I can distinguish animal abuse features”, 81.8% of the respondents agreed (Figure 1). Interestingly, in item 8 of Section 2, the majority of respondents stated that they had not acquired sufficient knowledge in veterinary training. The mean score of item 8 for respondents who answered that they could distinguish abuse features (3.17 ± 1.62 for animal abuse and 3.27 ± 1.62 for human abuse) was significantly higher than that of those who answered that they could not (2.62 ± 1.50 for animal abuse and 2.61 ± 1.49 for human abuse) (Table 5).

Approximately 45.7% of the respondents stated that dealing with animal abuse cases was beyond their profession or competence, and 76.1% of the respondents stated that they were unable to deal with human abuse. The results of the cross-analysis with item 8 also indicated that the more the respondents believed they had been provided with adequate training, the less they agreed that dealing with abuse cases was beyond their ability (Table 5).

Most respondents (78.9% for animal abuse and 80.2% for human abuse) were worried that dealing with abuse cases would irritate clients (Table 6). However, this issue was not significantly associated with item 8 of Section 2, “during veterinary training, I was provided with adequate information and training to identify and prevent animal abuse”, and only a borderline significant association was observed when dealing with animal abuse (*p* = 0.077) (Table 5). Moreover, there was no statistically significant difference between this issue and their choice of mandatory reporting of abuse cases (Table 6).

Moreover, most respondents (91.1% for animal abuse and 87.9% for human abuse) stated that reporting abuse cases was a civic responsibility (Table 6).

As shown in Table 6, those who stated that reporting animal abuse cases was their civic responsibility were significantly associated with those who stated that it should be mandatory to report animal abuse. Among those who agreed that reporting animal abuse should be mandatory, 94.5% stated that it was their civic responsibility to report animal abuse cases; even among those who did not agree to report animal abuse, 64.3% thought it was their civic responsibility to report abuse.

The comparison between the mandatory reporting of human abuse and whether the reporting of human abuse and animal abuse is a civic responsibility both showed significant associations (*p* < 0.01 and *p* = 0.029, respectively), as listed in Table 6. Among the respondents who agreed that reporting human abuse should be mandatory, the proportion of those who stated that reporting animal abuse is a civic responsibility (93.9%) and the proportion of those who stated that reporting human abuse is a civic responsibility (94.5%) was similar.

Respondents who stated that it should be mandatory to report animal abuse and that reporting animal abuse is a civic responsibility accounted for 83.8% of all respondents (207 out of 247), while respondents who stated that it should be mandatory to report human abuse and that reporting human abuse is a civic responsibility accounted for 66.4% of all respondents (164 out of 247).

#### 3.3.3. Willingness and Obligation Associated with Reporting Abuse Cases

With respect to the association between the willingness and the obligation to report animal abuse, among those who agreed with the mandatory reporting of animal abuse, there was a significantly higher percentage who would report animal abuse cases (*p* < 0.01) (Table 6). Among those who stated that it should not be mandatory to report animal abuse cases, the largest proportion chose to report only severe animal abuse cases (64.3%), and a few respondents chose not to report at all (14.3%). Although there was no significant association between whether it should be mandatory to report animal abuse cases and the willingness to report human abuse cases, the trend was similar.

In addition, among those who agreed that it should be mandatory to report human abuse, there was a significantly higher percentage who would report both animal abuse and human abuse (*p* = 0.032 and *p* < 0.01, respectively) (Table 6). Among those who stated that it should be mandatory to report human abuse cases, the largest proportion chose to report all abuse cases (54.9% for animal abuse and 56.7% for human abuse). Among those who stated that it should not be mandatory to report human abuse cases, the largest proportion chose to report only severe abuse cases (40.2% for animal abuse and 37.2% for human abuse).

#### 3.3.4. Demographic Characteristics and Respondents’ Management of Abuse Cases

With respect to the correlation between demographic characteristics and respondents’ attitudes toward mandatory reporting of abuse cases, there were no significant differences for gender, age, and year of graduation (Table 7).

In addition, we compared the differences in the thinking of students (after 2021) and veterinarians (before 2021) in this section. The results indicated that there were no significant differences between the two graduation status groups for these questions, except for the willingness to report both animal and human abuse cases (Table 8).

### 3.4. Sections 4 and 5: Physical and Mental Animal Abuse

Sections 4 and 5 were filled in by practicing veterinarians with clinical experience. In the raw data, some respondents claimed they did not have the knowledge to identify abuse cases, and therefore they had encountered no abuse cases clinically. Because this might mean that these respondents were unfamiliar with the definition of physical and mental animal abuse, we excluded these respondents. Comparisons of the raw and recalculated data are listed in Table 9.

Of the 119 veterinarians who claimed they had the knowledge to identify physical abuse, or who had encountered abuse cases, only 36.1% (43 out of 119) had not seen suspected physical abuse in the past five years. We obtained similar results (41 out of 113, 36.3%) for those who said they had encountered animal mental abuse cases in the past five years. Among these respondents, 110 believed they had the knowledge to identify both physical and mental abuse.

For these 119 veterinarians, we analyzed the correlation between their belief about whether they had the knowledge to identify physical abuse and the physical animal abuse cases they had suspected in the past five years. The results showed a significant correlation (*p* = 0.013). Conversely, in the analysis of 113 veterinarians who believed they had the knowledge to identify mental abuse, the result was insignificant (*p* = 0.429).

Our results showed male veterinarians were more likely to encounter physical abuse cases than females (*p* = 0.016). There were no significant differences between encountering physical animal abuse and other examined demographic categories, including age and year of graduation. If we combined these data with the average number of visiting cases per month, as asked in Section 1, the incidence of physical animal abuse encountered by the veterinarians was 0.19 cases per 100 patients.

Details of the abuse cases are listed in Table 10. With respect to, “who brought the abused animal to the veterinarian”, the most likely person was the owner, followed by other people. Dogs were the most common species being abused, followed by cats. Approximately 10% of the animal abuse cases co-occurred with suspected human abuse.

In the two sections, the reasons for suspecting animal abuse were also explored. The results for physical abuse are summarized in Table 11, while the results for mental abuse are summarized in Table 12.

The responses regarding the injured body parts and types of injury are presented in Table 13. Among these cases, the most commonly injured body parts were limbs and/or head, and the most common injury types were emaciation, malnutrition, or poor fur condition caused by varying degrees of neglect. Three cases in which the animals were evidently dead or had been eaten by other animals are noteworthy. There was also a mention by a carcass inspection veterinarian of inhumane slaughter of economic animals that were improperly stunned and exsanguinated.

We further analyzed whether physical abuse cases had been encountered in the past five years and veterinarians’ willingness to report them. The results are shown in Table 14, which shows that there was a significant difference among groups (*p* = 0.013). We found that veterinarians who chose to report all abuse cases had encountered fewer suspected cases of physical abuse in the past five years; by contrast, veterinarians who only reported severe cases had encountered many more physical abuse cases.

## 4. Discussion

### 4.1. Animal Abuse Cases Encountered by Veterinarians

In this study, the incidence of suspected physical animal abuse encountered by veterinarians in Taiwan was 0.16 cases per 100 patients. In the past five years, 63.9% of our respondents had seen suspected physical abuse. In the United States, the incidence of suspected animal abuse cases was reported as 0.56 cases per 100 patients [1]. In Australia, the incidence was 0.12 cases per 100 patients, and 92% of respondents had seen suspected abuse [15]. In New Zealand, 63% of respondents had seen suspected abuse [14], while 87% of respondents had seen suspected abuse in the United States [10]. In South Korea, 86.5% of respondents had seen suspected abuse [17]. Therefore, compared with those in other countries, Taiwanese veterinarians showed a very low probability of encountering suspected animal abuse cases. However, different methods of recording the frequency of animal abuse and different definitions of animal abuse mean that making meaningful comparisons between studies is difficult.

In addition, education level and personal cognition, resulting in inconsistent individual judgment standards, may cause data deviation. Therefore, the incidence of animal abuse may not reflect the actual situation in Taiwan. A previous study, which specifically underscored the need for education and training in a group of board-certified veterinary pathologists, pointed out the current situation of lacking associated knowledge for handling veterinary forensic cases [18]. In this study, respondents who believed they knew how to distinguish animal abuse features were correlated with the encounter of animal abuse. The fact that veterinarians in Taiwan generally evaluated veterinary forensic training courses as insufficient cannot be overlooked. One reason for veterinarians believing that they knew how to identify animal abuse might be that they had learned through experience; however, they might not have had a systematic understanding of the topic. Although empirical learning is indeed important, the lack of curricula in Taiwan’s veterinary training on how to identify animal abuse may be responsible for the low incidence of animal abuse.

In addition, it is worth noting that approximately 10% of the animal abuse cases co-occurred with suspected human abuse. Co-occurrence rates in other studies were 23.7% in Australia [15] and 56% in New Zealand [14]. Due to different definitions of abuse, the reported co-occurrence rate was 9 to 88%, according to one study in the United States [19]. Veterinarians are not professionally trained to detect human abuse; therefore, the co-occurrence rate may be underestimated.

In addition, we found that the frequency of encountering physical animal abuse was greater for male veterinarians than for females. There was no difference for age or year of graduation. In other studies, females [15,17] and younger [1,15,17] veterinarians had a higher frequency of encountering animal abuse. However, this trend was not observed in our results.

Similar across all surveys, dogs were the most commonly abused species encountered by veterinarians, followed by cats. This could be explained by the fact that as companion animals, humans have easy access to them. In addition, because they are relatively small, humans can easily control them. However, this result was also affected by the composition of veterinary practice types of the respondents.

In line with the results of other studies, the most common injury types in this study were emaciation, malnutrition, or poor fur condition caused by varying degrees of neglect (*n* = 49, 41.2%). The second most common injury type was bruises or hemorrhage (*n* = 41, 34.5%), followed by abrasions or lacerations. This result is consistent with those of previous studies [15]. Among the responses we received, one summarized the current situation in Taiwan for us: “The abuses often seen in animal hospitals are negligence: starvation, extreme dehydration, long-term exposure to the sun without shelter, dirty and covered in maggots, space constraints, performing obsessive-compulsive behavior on animals. Because the owner did not think these were abusive acts, the animals would be sent to the veterinarian only if there was an imminent danger. Too many cases were attributed to a long-term lack of quality of life. Most cases of obvious acts of violence would not be sent to veterinarians by the owner. Only when violent behavior causes death may animals be sent for forensic examination.” In Section 3, this respondent stated that he/she would report only severe cases when encountering animal abuse cases. This result reveals that veterinarians exposed to a large number of animal abuse cases might not report all of them. Most practicing veterinarians exposed to many cases of animal abuse chose to report only the severe cases. We also found that compared to veterinarians who had graduated, students who had yet to graduate were more likely to report all abuse cases. These changes in thinking may be related to the veterinarian’s practice environment, the impact of education, policy support, and/or practical considerations.

### 4.2. Attitudes toward Animal and Interpersonal Abuse

In this study, veterinarians generally agreed that animal abuse should be prevented and differed only with respect to the degree of personal willingness. Veterinarians’ motivation to interfere in animal abuse cases was positively related to their moral or legal responsibility, willingness to assist, and agreement with mandatory reporting. However, compared to animal abuse, veterinarians were significantly less willing to interfere in interpersonal violence. In addition, dealing with human abuse was considered to be beyond their ability and was not considered as much a civic responsibility as the former. We also found that approximately 80% of the respondents agreed that animal abuse and domestic abuse frequently co-occur, but this did not affect their willingness to report it. This is probably related to the traditional role of veterinarians in serving to promote animal health.

Veterinarians play a crucial role in animal welfare and public health. By dealing with animal abuse cases, veterinarians contribute to people’s overall health. However, a significant barrier faced by veterinarians is the fact that current legal and police systems are unable to ensure the safety and welfare of victims [17]. Other factors that are often discussed include insufficient training, damage to veterinarian–client relationships, loss of economic support, and a possible breach of personal safety.

Unexpectedly, 88.7% of our respondents thought it should be mandatory for veterinarians to report animal abuse cases, but only 66.5% felt the same about human abuse cases. Furthermore, those who were more willing to report animal abuse cases were more likely to agree that it should be mandatory to report animal abuse cases. A recent study [10] in the United States on whether reporting should be mandatory showed that respondent veterinarians strongly supported (24.0%) or supported (42.1%) these laws, with 19.5% having no opinion, 11.2% opposing, and 3.2% being strongly opposed to such laws. These data imply that veterinarians in Taiwan have a considerably high level of awareness regarding animal protection. We hope that these data lead to a revision of related laws. Minimal protection for veterinarians and animals could be initiated by modifying the law that immunizes veterinarians from civil and criminal liability. We also suggest putting in place an animal abuse tracking system to actively intervene and prevent animal abuse cases.

Our results also suggest that education may influence veterinarians’ attitudes when they are faced with abuse cases. Respondents who considered they had been provided with adequate training were more willing to deal with animal abuse and more capable of distinguishing abuse cases and did not feel that dealing with abuse cases was beyond their ability. These results were similar to those of a previous study [20], which established that specialized training equips veterinarians with the skills and confidence to report abuse. However, a large proportion of our respondents believed that the training provided was insufficient. Enhanced education and training could increase veterinarians’ confidence in communicating with clients and improve their ability to identify abuse cases and understand the notification process and other assistive resources that can be applied when encountering abuse cases.

### 4.3. Limitations

There were some limitations to this study. First, the process we used to collect data from the questionnaires made it impossible to guarantee that there were no repeat respondents or respondents who were not veterinarians. Second, the respondents’ definition of physical or mental abuse could not be assessed. Therefore, the differences in defining animal abuse could have led to response bias. Third, the low response rate and the fact that 40% of respondents are undergraduate veterinary students could influence the results. Last, the voluntary nature of the survey and the low possibility of the abused animal being sent to veterinary hospitals might cause statistical bias.

## 5. Conclusions

This is the first study in Taiwan to examine the current situation of animal abuse from the perspective of veterinarians and also the first study to collect the attitudes of veterinarians when dealing with abuse cases. Our results revealed that the incidence of suspected physical animal abuse encountered by veterinarians in Taiwan was 0.16 cases per 100 patients. Approximately 10% of animal abuse cases co-occur with suspected human abuse. This study and other research indicated that animal abuse and domestic abuse co-occur frequently. Hence, the traditional role of veterinarians should be enhanced through education for identifying and responding to animal cruelty. Increasing the sensitivity of veterinarians to animal abuse will enable early detection and tracking of animal abuse and further reduce the incidence of domestic abuse, thereby promoting animal welfare and public health. The fact that 88.7% of our respondents thought that veterinarians should mandatorily report animal abuse cases must not be ignored. However, mandatory reporting may cause pressure on veterinarians. Therefore, it is hoped and recommended that the relevant authorities modify the law to provide immunity to veterinarians from civil and criminal liability when reporting animal abuse cases.

## Figures and Tables

**Table 1:** Demographic and practice characteristics. (*n* = 247).

Demographic Characteristics	Number	(%)
**Gender**		
Male Female	86 161	34.8 65.2
**Age (years)**		
18–29 30–39 40–49 50–64 ≥65	161 52 18 16 0	65.2 21.1 7.3 6.4 0
**Year of graduation**		
After 2021 2016–2020 2011–2015 2001–2010 1991–2000 1981–1990 1971–1980 Before 1970	100 60 32 24 24 5 2 0	40.5 24.3 13.0 9.7 9.7 2.0 0.8 0
Type of clinical practice *		
Dogs and cats Small animals (except dogs and cats) Economic animals Exotic animals Researchers Government veterinarians Others	183 53 44 50 28 8 7	74.1 21.5 17.8 20.2 11.0 3.2 2.8

* Multiple choices were allowed.

**Table 2:** Attitude of veterinarians when faced with abuse cases.

Statement	Mean Score ± SD	Likert Scale					
		1	2	3	4	5	6
		Percentage of Responses					
**Item 1** When animal abuse is suspected, the veterinarian has a moral or legal responsibility to intervene.	5.05 ± 1.04	0.4	2.0	5.2	19.4	30.6	42.3
**Item 2** In practice, when presented with a suspected animal abuse case, I know my legal rights and responsibilities.	4.28 ± 1.56	6.5	10.9	10.9	20.6	22.6	28.6
**Item 3** In practice, when presented with an animal abuse case, I would assist in preventing it from happening again.	5.17 ± 0.97	0.4	1.2	4.8	13.3	34.7	45.6
**Item 4** People who abuse animals are more likely to abuse their children.	4.92 ± 1.28	2.0	3.2	8.5	21.4	17.3	47.6
**Item 5** People who abuse animals are more likely to abuse their spouse.	4.74 ± 1.35	2.8	4.0	11.3	21.4	19.8	40.7
**Item 6** People who abuse animals are more likely to commit other types of crime.	4.75 ± 1.43	2.4	6.5	12.1	17.3	16.1	45.6
**Item 7** People who train animals using punitive methods (physical correction, choker leash, electric collar, etc.) are more likely to abuse animals.	4.14 ± 1.38	3.6	10.9	14.9	28.2	23.4	19.0
**Item 8** During veterinary training, I was provided with adequate information and training to identify and prevent animal abuse.	3.07 ± 1.61	18.1	26.2	18.1	15.7	10.5	11.3
**Item 9** In practice, when domestic violence is suspected, the veterinarian has a moral responsibility to intervene.	3.85 ± 1.47	6.5	14.9	16.1	27.4	18.5	16.5
**Item 10** In practice, when presented with child or spousal abuse, I feel I should provide assistance to the client.	4.23 ± 1.41	3.2	10.5	15.7	23.0	25.0	22.6

Note: Mean scores and percentage of 6-point Likert scale from responses in Section 2 of the questionnaire in Appendix A (*n* = 247); 1 = strongly disagree, 2 = disagree, 3 = slightly disagree, 4 = slightly agree, 5 = agree, and 6 = strongly agree.

**Table 3:** Respondents’ demographic characteristics and their responses to the relationship between animal abuse and interpersonal violence and the intention to provide client assistance.

	Mean Score ± SD				
	Item 4	Item 5	Item 3	Item 10	Item 8
**Statement**	Animal abusers are more likely to…		I need to assist when there’s…		I was provided with adequate information in the veterinary training course.
	abuse children.	abuse spouse.	animal abuse.	child/spousal abuse.	
**Gender**					
Male Female	4.83 ± 1.44 4.97 ± 1.19	4.67 ± 1.50 4.77 ± 1.27	5.09 ± 1.09 5.22 ± 0.89	4.37 ± 1.46 4.16 ± 1.37	3.24 ± 1.69 2.98 ± 1.56
**Age (years)**					
18–29 30–39 40–49 50–64	4.80 ± 1.26 5.17 ± 1.26 4.83 ± 1.73 4.92 ±1.28	4.58 ± 1.34 ^a^ 5.08 ± 1.30 ^a^ 5.25 ± 0.93 ^a^ 4.74 ± 1.35 ^a^	5.28 ± 0.88 ^b^ 5.02 ± 1.02 ^b^ 4.72 ± 1.27 ^b^ 5.17 ± 0.97 ^b^	4.25 ± 1.35 4.06 ± 1.56 4.50 ± 1.34 4.38 ± 1.50	3.22 ± 1.57 2.77 ± 1.81 2.44 ± 1.10 3.25 ± 1.57
**Graduation year**					
After 2021 2016–2020 2011–2015 2001–2010 1991–2000 1981–1990 1971–1980	4.85 ± 1.28 4.72 ± 1.25 5.13 ± 1.23 5.33 ± 0.87 5.04 ± 1.57 4.80 ± 2.17 5.00 ± 0.00	4.62 ± 1.30 4.61 ± 1.29 4.90 ± 1.60 5.25 ± 0.90 4.79 ± 1.59 4.80 ± 2.17 5.00 ± 0.00	5.38 ± 0.91 5.00 ± 0.84 5.10 ± 1.08 5.21 ± 0.88 4.83 ± 1.34 5.20 ± 0.84 5.00 ± 0.00	4.26 ± 1.49 4.31 ± 1.18 4.10 ± 1.45 4.21 ± 1.53 4.33 ± 1.40 4.20 ± 1.64 2.00 ± 0.00	3.54 ± 1.60 ^c^ 2.90 ± 1.61 ^c^ 2.42 ± 1.54 ^c^ 2.71 ± 1.49 ^c^ 2.83 ± 1.37 ^c^ 3.20 ± 1.92 ^c^ 2.00 ± 0.00 ^c^
**Graduation year**					
After 2021 Before 2020	4.85 ± 1.28 4.97 ± 1.28	4.62 ± 1.30 4.82 ± 1.38	5.38 ± 0.91 ^d^ 5.03 ± 0.98 ^d^	4.26 ± 1.49 4.22 ± 1.35	3.54 ± 1.60 ^e^ 2.76 ± 1.53 ^e^

In this table, except for the borderline significant differences in groups ^a^ and ^b^ (group a, *p* = 0.052; group b, *p* = 0.064) and significant differences in groups ^c^, ^d^, and ^e^ (group c, *p* = 0.008; group d, *p* = 0.005; group e, *p* < 0.001), there were no significant differences in the other groups.

**Table 4:** A comparative analysis of veterinarians’ willingness to report abuse cases and veterinarians’ attitudes toward abuse cases.

		Mean Score ± SD			*p* Value
Statement	Target of Abuse	Not Report at All	Report Severe Cases	Report All Cases	
**Item 1** When animal abuse is suspected, the veterinarian has a moral or legal responsibility to intervene.	Animal Human	4.00 ± 0.77 4.44 ± 1.04	4.76 ± 1.12 4.94 ± 1.00	5.41 ± 0.81 5.28 ± 1.01	**<0.01** **<0.01**
**Item 9** In practice, when domestic violence is suspected, the veterinarian has a moral responsibility to intervene.	Animal Human	2.45 ± 1.29 2.44 ± 1.16	3.51 ± 1.38 3.60 ± 1.37	4.20 ± 1.41 4.40 ± 1.36	**<0.01** **<0.01**
**Item 3** In practice, when presented with an animal abuse case, I would assist in preventing it from happening again.	Animal Human	3.91 ± 1.38 4.24 ± 1.01	4.95 ± 0.91 5.06 ± 0.96	5.50 ± 0.82 5.49 ± 0.80	**<0.01** **<0.01**
**Item 10** In practice, when presented with child or spousal abuse, I feel I should provide assistance to the client.	Animal Human	3.36 ± 1.75 2.64 ± 1.29	3.92 ± 1.34 3.99 ± 1.29	4.61 ± 1.34 4.81 ± 1.19	**<0.01** **<0.01**
**Item 4** People who abuse animals are more likely to abuse their children.	Animal Human	4.73 ± 1.95 4.80 ± 1.55	4.96 ± 1.27 4.90 ± 1.30	4.89 ± 1.23 4.97 ± 1.21	0.804 0.822
**Item 5** People who abuse animals are more likely to abuse their spouse.	Animal Human	4.73 ± 1.95 4.32 ± 1.73	4.71 ± 1.41 4.72 ± 1.37	4.76 ± 1.25 4.84 ± 1.24	0.958 0.212

*p* Values < 0.05 were labeled in bold.

**Table 5:** Comparisons of mean scores of item 8 of Section 2 of the questionnaire in Appendix A (“During veterinary training, I was provided with adequate information and training to identify and prevent animal abuse”) among groups segregated by statement and target of abuse.

		Mean Score ± SD		*p* Value
Statement	Target of Abuse	No	Yes	
I can distinguish abuse features.	Animal Human	2.62 ± 1.50 2.61 ± 1.49	3.17 ± 1.62 3.27 ± 1.62	**0.037** **<0.01**
Dealing with abuse cases is beyond my profession or competence.	Animal Human	3.33 ± 1.54 3.64 ± 1.65	2.77 ± 1.64 2.89 ± 1.55	**<0.01** **<0.01**
I am worried that dealing with abuse cases will irritate clients.	Animal Human	3.42 ± 1.43 3.14 ± 1.46	2.98 ± 1.64 3.06 ± 1.64	0.077 0.734

*p* Values < 0.05 were labeled in bold.

**Table 6:** Comparisons between respondents who stated that it should be mandatory to report abuse cases and the consequences that they worried about and their attitude toward reporting abuse cases.

			It Should Be Mandatory to Report Animal Abuse.		
Statement	Target of Abuse	Belief (*n*, %)	No (*n*, %)	Yes (*n*, %)	*p* Value
Dealing with abuse cases is beyond my profession or competence.	Animal	No (134, 54.3%) Yes (113, 45.7%)	17 (60.7%) 11 (39.3%)	117 (53.4%) 102 (46.6%)	0.466
	Human	No (59, 23.9%) Yes (188, 76.1%)	6 (21.4%) 22 (78.6%)	53 (24.2%) 166 (75.8%)	0.746
			**It should be mandatory to report** **human abuse.**		
Dealing with abuse cases is beyond my profession or competence.	Animal	No (134, 54.3%) Yes (113, 45.7%)	51 (61.4%) 32 (38.6%)	83 (50.6%) 81 (49.4%)	0.106
	Human	No (59, 23.9%) Yes (188, 76.1%)	11 (13.3%) 72 (86.7%)	48 (29.3%) 116 (70.7%)	**<0.01**
			**It should be mandatory to report** **animal abuse.**		
I am worried that dealing with abuse cases will irritate clients.	Animal	No (52, 21.1%) Yes (195, 78.9%)	6 (21.4%) 22 (78.6%)	46 (21.0%) 173 (79.0%)	0.959
	Human	No (49, 19.8%) Yes (198, 80.2%)	6 (21.4%) 22 (78.6%)	43 (19.6%) 176 (80.4%)	0.823
			**It should be mandatory to report** **human abuse.**		
I am worried that dealing with abuse cases will irritate clients.	Animal	No (52, 21.1%) Yes (195, 78.9%)	20 (24.1%) 63 (75.9%)	32 (19.5%) 132 (80.5%)	0.404
	Human	No (49, 19.8%) Yes (198, 80.2%)	14 (16.9%) 69 (83.1%)	35 (21.3%) 129 (78.7%)	0.405
			**It should be mandatory to report** **animal abuse.**		
It is my civic responsibility to report abuse cases.	Animal	No (22, 8.9%) Yes (225, 91.1%)	10 (35.7%) 18 (64.3%)	12 (5.5%) 207 (94.5%)	**<0.01**
	Human	No (30, 12.1%) Yes (217, 87.9%)	7 (25.0%) 21 (75.0%)	23 (10.5%) 196 (89.5%)	0.057
			**It should be mandatory to report** **human abuse.**		
It is my civic responsibility to report abuse cases.	Animal	No (22, 8.9%) Yes (225, 91.1%)	12 (14.5%) 71 (85.5%)	10 (6.1%) 154 (93.9%)	**0.029**
	Human	No (30, 12.1%) Yes (217, 87.9%)	21 (25.3%) 62 (74.7%)	9 (5.5%) 164 (94.5%)	**<0.01**
			**It should be mandatory to report animal abuse.**		
Willingness to report abuse cases	Animal	Not report at all (11, 4.5%) Report severe cases (114, 46.2%) Report all cases (122, 49.4%)	4 (14.3%) 18 (64.3%) 6 (21.4%)	7 (3.2%) 96 (43.8%) 116 (53.0%)	**<0.01**
	Human	Not report at all (25, 10.1%) Report severe cases (107, 43.3%) Report all cases (115, 46.6%)	6 (21.4%) 12 (42.9%) 10 (35.7%)	19 (8.7%) 95 (43.4%) 105 (47.9%)	0.135
			**It should be mandatory to report human abuse.**		
Willingness to report abuse cases	Animal	Not report at all (11, 4.5%) Report severe cases (114, 46.2%) Report all cases (122, 49.4%)	3 (3.6%) 48 (57.8%) 32 (38.6%)	8 (4.9%) 66 (40.2%) 90 (54.9%)	**0.032**
	Human	Not report at all (25, 10.1%) Report severe cases (107, 43.3%) Report all cases (115, 46.6%)	15 (18.1%) 46 (55.4%) 22 (26.5%)	10 (6.1%) 61 (37.2%) 93 (56.7%)	**<0.01**

*p* Values < 0.05 were labeled in bold.

**Table 7:** Respondents’ demographic characteristics and their attitude toward whether it should be mandatory to report abuse cases.

Statement	It Should Be Mandatory to Report Abuse Cases When Veterinarians Encounter Deliberate abuse.					
	Target of Abuse: Animal			Target Of Abuse: Human		
Characteristics (*n*, %)	No (*n*, %)	Yes (*n*, %)	*p* Value	No (*n*, %)	Yes (*n*, %)	*p* Value
**Gender**			0.343			0.977
Male (86, 34.8%) Female (161, 65.2%)	12 (14.0%) 16 (9.9%)	74 (86.0%) 145 (90.1%)		29 (33.7%) 54 (33.5%)	57 (66.3%) 107 (66.4%)	
**Age (years)**			0.740			0.526
18–29 (161, 65.2%) 30–39 (52, 21.1%) 40–49 (18, 7.3%) 50–64 (16, 6.5%)	17 (10.6%) 7 (13.5%) 3 (16.7%) 1 (6.3%)	144 (89.4%) 45 (86.5%) 15 (83.3%) 15 (93.8%)		57 (35.4%) 16 (30.8%) 7 (38.9%) 3 (18.8%)	104 (64.6%) 36 (69.2%) 11 (61.1%) 13 (81.3%)	
**Graduation year**			0.836			0.412
After 2021 (100, 40.5%) 2016–2020 (61, 24.7%) 2011–2015 (31, 12.6%) 2001–2010 (24, 9.7%) 1991–2000 (24, 9.7%) 1981–1990 (5, 2.0%) 1971–1980 (2, 0.8%)	10 (10.0%) 8 (13.1%) 3 (9.7%) 4 (16.7%) 3 (12.5%) 0 (0.0%) 0 (0.0%)	90 (90.0%) 53 (86.9%) 28 (90.3%) 20 (83.3%) 21 (87.5%) 5 (100.0%) 2 (100.0%)		34 (34.0%) 21 (34.4%) 10 (32.3%) 9 (37.5.6%) 9 (37.5%) 0 (0.0%) 0 (0.0%)	66 (66.0%) 40 (65.6%) 21 (67.7%) 15 (62.5%) 15 (62.5%) 5 (100.0%) 2 (100.0%)	

**Table 8:** A comparative analysis of management of abuse and veterinarians’ graduation status.

			Veterinarians’ Graduation Status		
Statement	Target of Abuse	Belief (*n*, %)	After 2021 (*n*, %)	Before 2020 (*n*, %)	*p* Value
I can distinguish abuse features.	Animal	No (45, 18.2%) Yes (202, 81.8%)	22 (22.0%) 78 (78.0%)	23 (15.6%) 124 (84.4%)	0.204
	Human	No (75, 30.4%) Yes (172, 69.6%)	26 (26.0%) 74 (74.0%)	49 (33.3%) 98 (66.7%)	0.219
Dealing with abuse cases is beyond my profession or competence.	Animal	No (134, 54.3%) Yes (113, 45.7%)	59 (59.0%) 41 (41.0%)	75 (51.0%) 72 (49.0%)	0.217
	Human	No (59, 23.9%) Yes (188, 76.1%)	28 (28.0%) 72 (72.0%)	31 (21.1%) 116 (78.9%)	0.211
I am worried that dealing with abuse cases will irritate clients.	Animal	No (52, 21.1%) Yes (195, 78.9%)	25 (25.0%) 75 (75.0%)	27 (18.4%) 120 (81.6%)	0.209
	Human	No (49, 19.8%) Yes (198, 80.2%)	24 (24.0%) 76 (76.0%)	25 (17.0%) 122 (83.0%)	0.176
It is my civic responsibility to report abuse cases.	Animal	No (22, 8.9%) Yes (225, 91.1%)	7 (7.0%) 93 (93.0%)	15 (10.2%) 132 (89.8%)	0.386
	Human	No (30, 12.1%) Yes (217, 87.9%)	12 (12.0%) 88 (88.0%)	18 (12.2%) 129 (87.8%)	0.954
It should be mandatory to report abuse cases when veterinarians encounter deliberate abuse.	Animal	No (28, 11.3%) Yes (219, 88.7%)	10 (10.0%) 90 (90.0%)	18 (12.2%) 129 (87.8%)	0.585
	Human	No (83, 33.6%) Yes (164, 66.4%)	34 (34.0%) 66 (66.0%)	49 (33.3%) 98 (66.7%)	0.913
		**Willingness (*n*, %)**			
Willingness to report abuse cases	Animal	Not report at all (11, 4.5%) Report severe cases (114, 46.2%) Report all cases (122, 49.4%)	2 (2.0%) 34 (34.0%) 64 (64.0%)	9 (6.1%) 80 (54.4%) 58 (39.5%)	**<0.01**
	Human	Not report at all (25, 10.1%) Report severe cases (107, 43.3%) Report all cases (115, 46.6%)	7 (7.0%) 36 (36.0%) 57 (57.0%)	18 (12.2%) 71 (48.3%) 58 (39.5%)	**0.022**

*p* Values < 0.05 were labeled in bold.

**Table 9:** Frequency of suspected physical and mental animal abuse cases encountered by veterinarians in the past five years in Taiwan.

Average Frequency of Encounters in a Year	Raw Number (%)		Number Possessing Knowledge (%)	
	Physical Abuse	Mental Abuse	Physical Abuse	Mental Abuse
No abuse case was found	57 (42.9%)	61 (45.9%)	43 (36.1%)	41 (36.3%)
≤1 time	31 (23.0%)	32 (24.1%)	31 (26.1%)	32 (28.3%)
2–3 times	33 (24.8%)	32 (24.1%)	33 (27.7%)	32 (28.3%)
4–11 times	7 (5.3%)	5 (3.8%)	7 (5.9%)	5 (4.4%)
>11 times	5 (4.0%)	3 (2.2%)	5 (4.2%)	3 (2.7%)
**Total**	**133**	**133**	**119**	**113**

Note: Raw number indicates that practicing veterinarians (*n* = 133) believed they had encountered animal abuse case(s); the number possessing knowledge indicates among veterinarians who considered they had the knowledge to identify physical abuse cases or who had encountered abuse case(s).

**Table 10:** Who brought physically abused animals to the veterinarian, what species of abused animal was involved, and whether interpersonal violence was also involved in the animal abuse case. The results were calculated only from the answers of 119 veterinarians who claimed they had the knowledge to identify physical abuse, or who had encountered abuse cases.

Statement	Number (%)
Who brought the abused animal to the veterinarian? *	
Owner Others Public institutions (government veterinarians, animal protection officers, police, etc.)	61 (51.3%) 34 (28.6%) 15 (12.6%)
Private groups	19 (16.0%)
What species was the abused animal? *	
Dog Cat Small animals (except dogs and cats) Economic animal Exotic animal Laboratory animal ^	61 (51.3%) 53 (44.5%) 12 (10.1%) 7 (5.9%) 6 (5.0%) 1 (0.8%)
**Was suspected human abuse also involved in the animal abuse case?**	
No abuse Suspected abuse Known abuse No idea	72 (60.5%) 9 (7.6%) 1 (0.8%) 37 (31.1%)

* Multiple choices were allowed. ^ Options added by respondents.

**Table 11:** Reasons for suspecting the case to be physical animal abuse. Multiple choices were allowed for this question. This was an open-ended question, and the respondents could add options themselves. The results were calculated only from the answers of 119 veterinarians who claimed they had the knowledge to identify physical abuse, or who had encountered abuse cases.

Statement	Number (%)
Nature of injury	56 (47.1%)
Neglect	52 (43.7%)
Owner’s behavior	36 (30.3%)
Repeated presentation of injuries	21 (17.6%)
Inconsistent medical history	20 (16.8%)
Exposed or seen by witnesses	18 (15.1%)
Witness at clinic ^	1 (0.8%)
Inhumane slaughter	1 (0.8%)

^ Options added by respondents.

**Table 12:** Reasons for suspecting the case to be mental animal abuse. Multiple choices were allowed for this question. This was an open-ended question, and the respondents could add options themselves. The results were calculated only from the answers of 113 veterinarians who claimed they had the knowledge to identify mental abuse, or who had encountered abuse cases.

Statement	Number (%)
Alert, easily frightened	42 (37.2%)
Trembling, curl up	35 (31.0%)
Anxiety	29 (25.7%)
Aggressive	27 (23.0%)
Self-harm and compulsive behavior ^	1 (0.9%)

^ Options added by respondents. We listed the mental animal abuse table here because it could reflect the interaction situation when animals were abused; however, we did not perform further statistical analyses based on these data.

**Table 13:** The injured body part and type of injury caused by physical abuse. Multiple choices were allowed for these questions. These were open-ended questions, and the respondents could add options themselves.

Statement	Number (%)
**Injured body part**	
Limb injury Head injury Sternum, ribs, or vertebra injury Ocular injury or visual impairment Tail injury Genital injury Teeth injury Ear and auricular impairment Abdominal injuries (abdominal trauma or organ hemorrhage) ^ Pelvic fracture ^ Improper stun and exsanguination ^	50 (42.0%) 31 (26.1%) 26 (21.8%) 16 (13.4%) 10 (8.4%) 6 (5.0%) 5 (4.2%) 2 (1.7%) 2 (1.7%) 1 (0.8%) 1 (0.8%)
**Type of injury**	
Emaciation, malnutrition, or poor fur condition Bruises or hemorrhage Abrasions or lacerations Sharp force injuries (incised or stab wounds, etc.) Poisoning Thermal burn (chemical or heat, etc.) Animal fighting (dogfighting, etc.) Friction burn Asphyxia Fracture or dislocation ^ Neurological signs (carrying) ^ Clamped by traps or tied with rubber bands ^ Firearm injuries ^	49 (41.2%) 41 (34.5%) 29 (24.4%) 28 (23.5%) 16 (13.4%) 13 (10.9%) 13 (10.9%) 9 (7.6%) 5 (4.2%) 4 (3.4%) 1 (0.8%) 1 (0.8%) 1 (0.8%)

^ Options added by respondents.

**Table 14:** Comparisons between the suspected physical animal abuse cases encountered by veterinarians and their attitude toward reporting suspected animal abuse.

Average Frequency of Encounter in a Year	Not Report at All	Report Severe Cases	Report All Cases
No abuse case was recorded	1 (14.3%)	15 (24.2%)	25 (52.1%)
≤1 time	2 (28.6%)	21 (33.9%)	8 (16.7%)
2–3 times	1 (14.3%)	21 (33.9%)	11 (22.9%)
4–11 times	2 (28.6%)	4 (6.5%)	1 (2.1%)
>11 times	1 (14.3%)	1 (1.6%)	3 (6.3%)
**Total**	**7**	**62**	**48**

Note: There was a significant difference (*p* = 0.013).

## Data Availability

The data presented in this study are available on request from the corresponding author.

## Appendix A. Questionnaire

(The questionnaire in Chinese is also available online at https://forms.gle/iB7BRfNGrwea8Dj4A (accessed on 17 March 2022)).

Section 1: Demographics and practice characteristics

1. Gender
  - Male
  - Female
2. Age (years)
  - 18–29
  - 30–39
  - 40–49
  - 50–64
  - ≥65
3. Year of graduation
  - After 2021
  - 2016–2020
  - 2011–2015
  - 2001–2010
  - 1991–2000
  - 1981–1990
  - 1971–1980
  - Before 1970
4. Type of clinical practice (Multiple choices were allowed.)
  - Dogs and cats
  - Small animals (except dogs and cats)
  - Economic animals
  - Exotic animals
  - Researchers
  - Government veterinarians
  - Others
5. Average number of visiting cases per month
  - 0
  - 1–30
  - 31–60
  - 61–100
  - 100–200
  - >200

Section 2: Attitudes (6-point Likert scale from 1 to 6, 1 = strongly disagree, 2 = disagree, 3 = slightly disagree, 4 = slightly agree, 5 = agree, and 6 = strongly agree)

Item 1. When animal abuse is suspected, the veterinarian has a moral or legal responsibility to intervene.
Item 2. In practice, when presented with a suspected animal abuse case, I know my legal rights and responsibilities.
Item 3. In practice, when presented with an animal abuse case, I would assist in preventing it from happening again.
Item 4. People who abuse animals are more likely to abuse their children.
Item 5. People who abuse animals are more likely to abuse their spouse.
Item 6. People who abuse animals are more likely to commit other types of crime.
Item 7. People who train animals using punitive methods (physical correction, choker leash, electric collar, etc.) are more likely to abuse animals.
Item 8. During veterinary training, I was provided with adequate information and training to identify and prevent animal abuse.
Item 9. In practice, when domestic violence is suspected, the veterinarian has a moral responsibility to intervene.
Item 10. In practice, when presented with child or spousal abuse, I feel I should provide assistance to the client.

Section 3: Management of abuse

Q1. Indicate your willingness to report suspected animal and human abuse.
  - Target of abuse: Animal
    🙢 Not report at all
    🙢 Report severe cases
    🙢 Report all cases
  - Target of abuse: Human
    🙢 Not report at all
    🙢 Report severe cases
    🙢 Report all cases
Q2. It is my civic responsibility to report abuse cases.
  - Target of abuse: Animal
    🙢 Yes
    🙢 No
  - Target of abuse: Human
    🙢 Yes
    🙢 No
Q3. I can distinguish abuse features.
  - Target of abuse: Animal
    🙢 Yes
    🙢 No
  - Target of abuse: Human
    🙢 Yes
    🙢 No
Q4. Dealing with abuse cases is beyond my profession or competence.
  - Target of abuse: Animal
    🙢 Yes
    🙢 No
  - Target of abuse: Human
    🙢 Yes
    🙢 No
Q5. I am worried that dealing with abuse cases will irritate clients (i.e., the owner).
  - Target of abuse: Animal
    🙢 Yes
    🙢 No
  - Target of abuse: Human
    🙢 Yes
    🙢 No
Q6. It should be mandatory to report abuse cases when veterinarians encounter deliberate abuse.
  - Target of abuse: Animal
    🙢 Yes
    🙢 No
  - Target of abuse: Human
    🙢 Yes
    🙢 No

Section 4: Physical animal abuse (limited to veterinary practitioners with clinical experience)

Q1. I believe I have the knowledge to identify suspected animal abuse.
  - Yes
  - No
Q2. Frequency of suspected animal abuse cases I have encountered within the past five years
  - No abuse case was found
  - ≤1 time
  - 2–3 times
  - 4–11 times
  - >11 times
Q3. Who brought the abused animals to you? (Multiple choices were allowed.)
  - Owner
  - Others
  - Public institutions (government veterinarians, animal protection officers, police, etc.)
  - Private groups
Q4. Reasons for suspecting the case to be physical animal abuse (Multiple choices were allowed. Open-ended question.)
  - Nature of injury
  - Exposed or seen by witnesses
  - Owner’s behavior
  - Inconsistent medical history
  - Repeated presentation of injuries
  - Neglect
Q5. What was the species of the abused animal? (Multiple choices were allowed. Open-ended question.)
  - Dog
  - Cat
  - Small animals (except dogs and cats)
  - Economic animal
  - Exotic animal
Q6. Injured body part caused by physical abuse. (Multiple choices were allowed. Open-ended question.)
  - Head injury
  - Ocular injury or visual impairment
  - Ear and auricular impairment
  - Teeth injury
  - Sternum, ribs, or vertebra injury
  - Limb injury
  - Genital injury
  - Tail injury
Q7. Type of injury caused by physical abuse. (Multiple choices were allowed. Open-ended question.)
  - Poisoning
  - Thermal burn (chemical or heat, etc.)
  - Friction burn
  - Asphyxia
  - Sharp force injuries (incised or stab wounds, etc.)
  - Bruises or hemorrhage
  - Abrasions or lacerations
  - Animal fighting (dogfighting, etc.)
  - Emaciation, malnutrition, or poor fur condition
Q8. Was suspected human abuse also involved in the animal abuse case?
  - No abuse
  - Suspected abuse
  - Known abuse
  - No idea

Section 5: Mental animal abuse (limited to veterinary practitioners who had the clinical experience)

Q1. I believe I have the knowledge to identify suspected animal abuse.
  - Yes
  - No
Q2. Frequency of suspected animal abuse cases I have encountered within the past five years
  - No abuse case was found
  - ≤ 1 time
  - 2–3 times
  - 4–11 times
  - > 11 times
Q3. Who brought the abused animals to you? (Multiple choices were allowed.)
  - Owner
  - Others
  - Public institutions (government veterinarians, animal protection officers, police, etc.)
  - Private groups
Q4. Reasons for suspecting the case to be physical animal abuse (Multiple choices were allowed. Open-ended question.)
  - Trembling, curl up
  - Anxiety
  - Aggressive
  - Alert, easily frightened
Q5. What was the species of the abused animal? (Multiple choices were allowed. Open-ended question.)
  - Dog
  - Cat
  - Small animals (except dogs and cats)
  - Economic animal
  - Exotic animal
Q6. Was suspected human abuse also involved in the animal abuse case?
  - No abuse
  - Suspected abuse
  - Known abuse
  - No idea
